# The cervical blood patch: A therapeutic “miraculous” for cerebrospinal fluid leaks: A case report

**DOI:** 10.1097/MD.0000000000037035

**Published:** 2023-02-02

**Authors:** Sami Kaan Coşarcan, Ömür Erçelen

**Affiliations:** a VKV American Hospital, Department of Anesthesiology, Istanbul, Turkey; b Koç University, School of Medicine, Department of Anesthesiology and Reanimation, Istanbul, Turkey; c VKV American Hospital, Department of Anesthesiology and Algology Clinic, Istanbul, Turkey.

**Keywords:** bone spur, cervical osteophytes, CSF leaks, epidural blood patch, headache, pain management

## Abstract

**Rationale:**

Cerebrospinal fluid (CSF) leaks, arising from abnormal openings in the protective layers surrounding the spinal cord and brain, are a significant medical concern. These leaks, triggered by various factors including trauma, medical interventions, or spontaneous rupture, lead to the draining of CSF—an essential fluid safeguarding the nervous system. A classic symptom of CSF leaks is an incapacitating headache exacerbated by sitting or standing but relieved by lying down. Spontaneous intracranial hypotension (SIH) denotes the clinical condition marked by postural headaches due to spontaneous CSF leakage and hypotension, often misdiagnosed or underdiagnosed. While orthostatic headaches are the hallmark, SIH may manifest with an array of symptoms including nausea, tinnitus, hearing loss, visual disturbances, and dizziness. Treatment options encompass conservative measures, epidural blood patches (EBP), and surgery, with EBP being the primary intervention.

**Patient Concern:**

The patient did not express any specific concerns regarding their medical diagnosis. However, they did harbor apprehensions that their condition might necessitate surgical intervention in the future.

**Diagnosis:**

The patient had been treated with antibiotics with a pre-diagnosis of sinusitis and was admitted to the neurology department of our hospital when his symptoms failed to improve. Cranial magnetic resonance imaging was interpreted as thickening of the dural surfaces and increased contrast uptake, thought to be due to intracranial hypotension. Cranial MR angiography was normal. Full-spine magnetic resonance imaging revealed a micro-spur at the C2 to 3 level and the T1 to 2 level in the posterior part of the corpus.

**Interventions:**

The cervical EBP was performed in the prone position under fluoroscopic guidance. There were no complications.

**Outcomes:**

The patient was invited for follow-up 1 week after the procedure, and control examination was normal.

**Lessons:**

SIH poses a diagnostic challenge due to its diverse clinical presentation and necessitates precise imaging for effective intervention. Cervical EBP emerges as a promising treatment modality, offering relief and improved quality of life for individuals grappling with this condition. However, clinicians must carefully assess patients and discuss potential risks and benefits before opting for cervical blood patches.

## 1. Introduction

Cerebrospinal fluid (CSF) leaks occur in case of an abnormal opening in the layers of protective tissue surrounding the spinal cord and brain. This opening can lead to the leakage of CSF, which cushions and protects the nervous system. Such leaks can result from various causes, including trauma, medical procedures, or spontaneous rupture due to underlying conditions. The hallmark symptom of a CSF leak is a debilitating headache that often worsens when sitting or standing and that improves when lying down. The term spontaneous intracranial hypotension (SIH) refers to a clinical condition characterized by debilitating postural headaches secondary to spontaneous CSF leakage and/or CSF hypotension.^[1]^ SIH is frequently misdiagnosed or underdiagnosed. Estimates suggest that SIH is not uncommon, with a 5-year incidence of 100,000 individuals per year, half that of subarachnoid hemorrhage.^[1,2]^ The most common symptom is an orthostatic headache, which usually worsens in the upright position and improves in the supine position. Neuroimaging plays an important role in the diagnosis and monitoring of SIH. Spine imaging aims to classify and localize the site of the CSF leak. Pinpointing the exact location of the leak is essential for successful treatment, which includes targeted epidural patching and surgical closure when conservative measures fail to provide long-term relief.^[2,3]^ The variability of SIH is also reflected in the wide range of signs and symptoms observed at presentation. Although headache is the most common symptom, orthostatic headache, once considered an essential feature of SIH, is not always present. Other signs/symptoms include nausea/vomiting, tinnitus, vertigo, hearing loss, photophobia, other visual symptoms, diplopia, and dizziness.^[4]^ CSF leaks are currently regarded as occurring through 3 main mechanisms: meningeal diverticula, ventral dural tears, and CSF-venous fistulas (CVFs). Diverticula were the most common findings (42%) in 1 large series study, followed by ventral dural tears (27%) and CVFs (3%), the remaining 28% cases being of undetermined cause.^[5]^ Since the publication of this series, CVFs have been increasingly detected, and it appears likely that their prevalence will rise as techniques capable of identifying them more reliably emerge. Brain magnetic resonance imaging (MRI) with contrast is the most sensitive single imaging test for diagnosing SIH and should be the first test performed. The findings of SIH at brain MRI all essentially derive from the same underlying problem of low CSF volume.^[4,6]^ Treatment options for SIH include conservative therapy, epidural blood patch (EBP), and surgery. Conservative therapy for SIH consists of bed rest, oral hydration, and oral caffeine administration. No headache medication has been reported to be widely effective in the treatment of SIH. EBP is the most common intervention for CSF leaks. Surgery is generally reserved for patients with well-localized CSF leaks who fail to benefit from EBP.^[4,6,7]^

The purpose of this article is to discuss the “miraculous” effect of cervical EBP in the treatment of intracranial hypotension caused by cervical osteophytes.

## 2. Patient concern

The patient did not express any specific concerns regarding their medical diagnosis. However, they did harbor apprehensions that their condition might necessitate surgical intervention in the future. The patient conveyed satisfaction with the explanations provided by the departments of algology and neurology, affirming a comprehensive understanding of the treatment algorithm. Nonetheless, their apprehension centered on potential adverse effects associated with the cervical EBP procedure. The patient explicitly indicated their acceptance of the treatment and associated risks, while meticulously weighing the anticipated benefits against any potential compromise to their overall quality of life. Specifically, they expressed concerns regarding the likelihood of a resurgence of headaches post-procedure and the possibility of treatment ineffectiveness. No financial or communication concerns were reported.

## 3. Diagnosis

**Figure 1.**
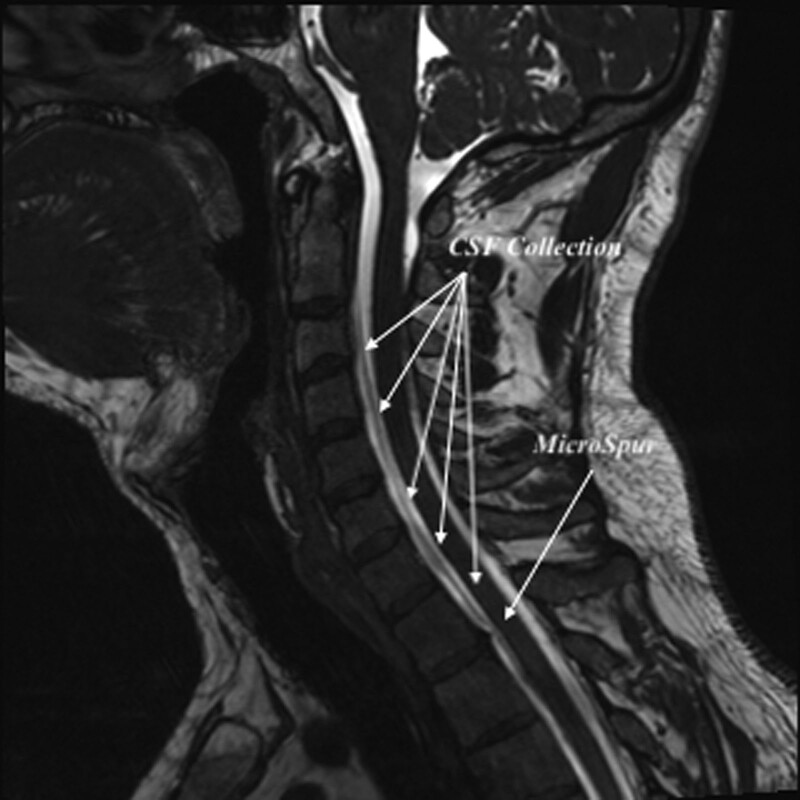
Full-spine magnetic resonance imaging.

**Figure 2.**
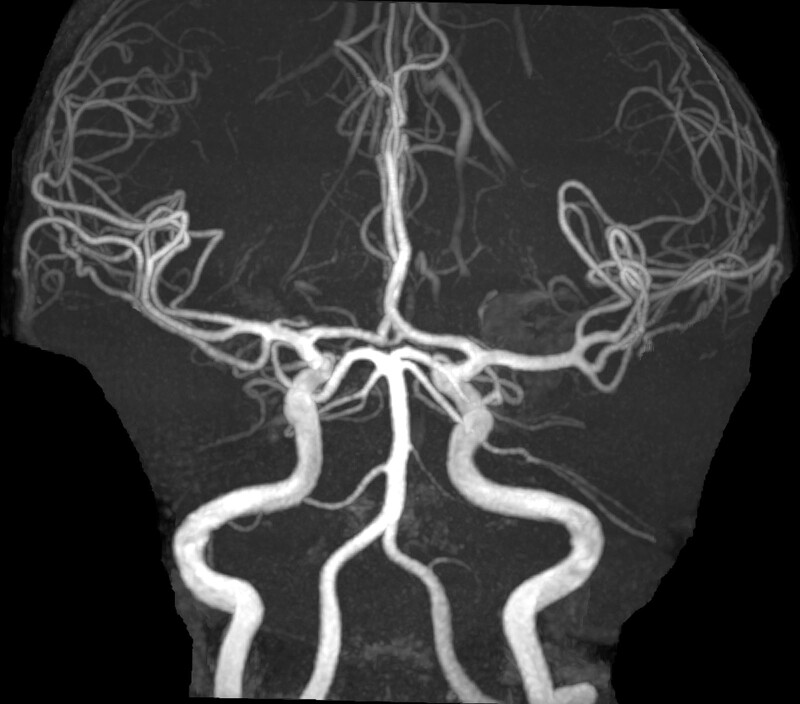
Cranial magnetic resonance angiography image.

A 39-year-old man was seen by an otolaryngologist at another hospital due to his headaches persisting despite non-steroidal anti-inflammatory drug use and presence of nausea. The patient had been treated with antibiotics with a pre-diagnosis of sinusitis and was admitted to the neurology department of our hospital when his symptoms failed to improve. Neurological examination performed in our neurology clinic was unremarkable. The headache was positional, increasing in intensity when standing and walking and decreasing when lying down and resting. Cranial MRI was interpreted as thickening of the dural surfaces and increased contrast uptake, thought to be due to intracranial hypotension. Full-spine MRI revealed a micro-spur at the C2 to 3 level and the T1 to 2 level in the posterior part of the corpus. A 5-mm CSF collection was observed at the thickest part of the anterior epidural space at the C2-T1 interval, and a 1-mm CSF collection at the posterior epidural space at the thoracic level (Fig. 1A and B). Cranial MR angiography was normal (Fig. 2). The patient was then admitted to the neurology clinic and started on intravenous hydration and analgesia. The algology clinic was consulted, and it was decided to perform a cervical EBP. The patient was examined at the algology clinic, where a positional headache compatible with intracranial hypotension was noted. Neurological examination was normal. Coagulation parameters were analyzed in the laboratory, and no problems were detected.

## 4. Interventions

**Figure 3.**
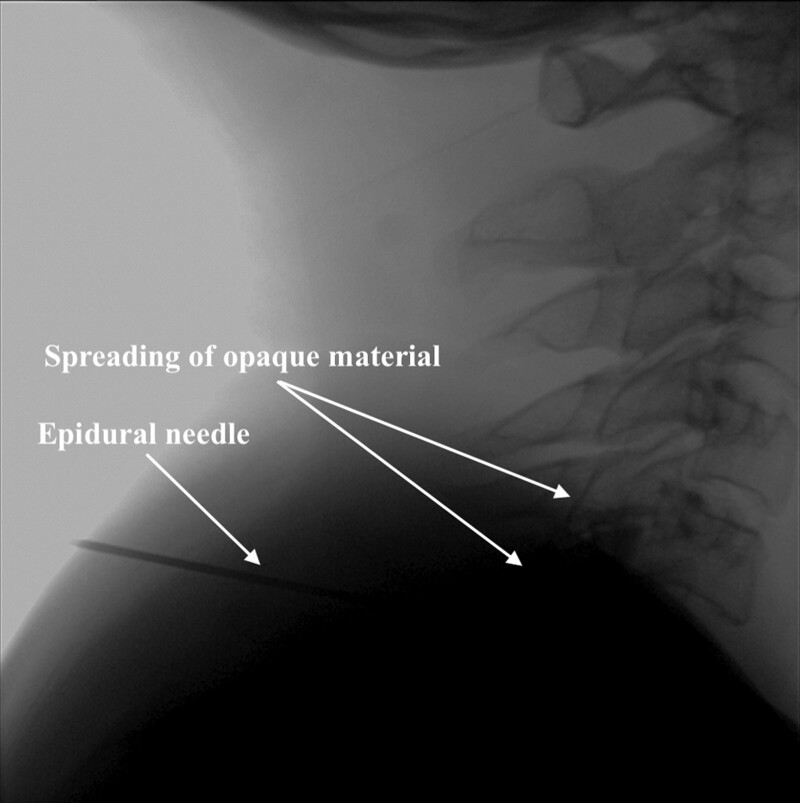
Placement of cervical epidural needle under fluoroscopic guidance and spreading of opaque material.

Once written informed consent had been obtained, the patient was taken to the operating room for a blood patch procedure. Mild sedation was administered. The epidural space was entered with an 18 G needle at the C6 to 7 level under fluoroscopy, with the patient in the prone position. The epidural space was confirmed using the loss of resistance technique. Epidural spread into the epidural space was confirmed by administering 2 mL of opaque material through an epidural Tuohy needle (Fig. 3). Ten milliliters of blood were collected from the right antecubital vein under sterile conditions, 7 mL of which were injected into the epidural space. Thirty minutes after the procedure, the headache was completely resolved. The patient was observed for 24 hours post-procedurally. No complications were noted, and he was discharged.

## 5. Outcomes

The patient was invited for follow-up 1 week after the procedure, and control examination was normal. He reported only 2 headaches, both relieved by paracetamol, within that 1-week period. The patient returned to a normal working life and expressed a very high level of satisfaction. He was evaluated via telemedicine 1, 3, and 6 months subsequently, and reported no longer experiencing headaches or nausea and being very satisfied with his life.

## 6. Discussion

SIH is caused by leakage of CSF from the spine. Although CSF leaks can occur from the base of the skull, these types of leaks do not typically cause orthostatic headache, are more commonly associated with high (rather than low) CSF pressure, and do not cause the brain imaging manifestations of SIH. CSF leaks are currently known to occur through 3 main mechanisms: meningeal diverticula, ventral dural tears, and CVFs. Diverticula were the most common (42%) findings in 1 large case series, followed by ventral dural tears (27%), and CVFs (3%), the remaining 28% being of undetermined cause.^[3,8,9]^ In our case, the intracranial hypotension was caused by microspores associated with disrupted dural surfaces.

SIH can be characterized by a wide range of symptoms, the most common being headache (positional), nausea, vomiting, neck pain, hearing problems, and visual disturbances. Headaches are more common in the diffuse type but can also be concentrated in the occipital region.^[3,10]^ In our case, the positional headache was diffuse in character, and the other accompanying symptom was nausea. The patient also complained of occasional neck pain but had no other accompanying complaints.

Conservative methods (bed rest, hydration, analgesics, caffeine, steroids, etc), blood patch, and surgery (repair of dural defects, CSF fistulae, etc) are used in treatment. However, the success rate of conservative treatment in published studies is quite low. A single EBP has been shown to exhibit a success rate of approximately 60%. Comparisons of high and low volume EBP have reported a significant positive success rate in high volume patients.^[3,4,11,12]^ Targeted EBP is a specialized form of EBP that aims to precisely target the identified CSF leak site. This procedure can be performed under fluoroscopy or computed tomography guidance, thus enhancing its effectiveness.^[11,12]^ In our case, conventional treatments failed to provide adequate results. Based on the results of the spinal MRI, we applied a targeted blood patch after determining the level of CSF leakage.

The injected blood forms a clot that acts as a sealant over the site of the CSF leak. This clot helps stop ongoing leakage of CSF. The blood volume introduced into the epidural space creates pressure, which can help seal the leak. It also increases the pressure in the intracranial space, which can reduce the flow of CSF and further prevent leakage. The blood clot can also stimulate the body natural healing processes. Scar tissue may gradually form around the clot, reinforcing the seal and preventing future leaks.^[5,13]^ The optimal blood volume for a cervical blood patch may vary depending on the individual case and the location and size of the CSF leak. The procedure can be performed as a high-volume or low-volume procedure usually depending on the physician assessment of the patient condition. A small amount of blood (e.g. 5–10 mL) may be sufficient to seal a CSF leak, especially if this is small or in a relatively accessible location. Using a smaller volume of blood may reduce the risk of complications such as over-pressurization of the intracranial space.^[3–5]^ In our case, the CSF leak was limited to the cervical region. We decided to apply a cervical blood patch closest to the microspore level, the main source of leakage. We applied a targeted cervical blood patch at a small volume of 7 mL under fluoroscopy. Following application of the blood patch, we observed a rapid improvement in symptoms and no complications. We also achieved long-term improvement, probably due to healing by scarring, one of the known mechanisms of action.

## 7. Conclusion

Cervical blood patches are generally considered safe and effective for treating intracranial hypotension. However, as with any medical procedure, there are some risks, including infection, bleeding, and nerve injury. The decision to use a cervical patch should be made after careful assessment of the patient condition and discussion of the potential risks and benefits with the healthcare provider.

## Author contributions

**Conceptualization:** Sami Kaan Coşarcan, Ömür Erçelen.

**Data curation:** Sami Kaan Coşarcan.

**Formal analysis:** Sami Kaan Coşarcan.

**Investigation:** Sami Kaan Coşarcan, Ömür Erçelen.

**Methodology:** Sami Kaan Coşarcan.

**Project administration:** Sami Kaan Coşarcan.

**Supervision:** Ömür Erçelen.

**Validation:** Sami Kaan Coşarcan.

**Writing – original draft:** Sami Kaan Coşarcan.

**Writing – review & editing:** Ömür Erçelen.
